# Unveiling Turbulence-Induced Stress Dynamics in Dented Pipe Using Acoustic Emission and Time–Frequency Analysis

**DOI:** 10.3390/s25237127

**Published:** 2025-11-21

**Authors:** Syed Muhamad Firdaus, Mazian Mohammad, Abdul Rahim Othman, Mohd Faridz Mod Yunoh

**Affiliations:** 1 Institute of Sustainable Energy & Resources (ISER), Universiti Teknologi PETRONAS, Seri Iskandar 32610, Malaysia; 2 Mechanical Engineering Department, Universiti Teknologi PETRONAS, Seri Iskandar 32610, Malaysia

**Keywords:** dented pipe, acoustic emission, computational fluid dynamic, Morlet wavelet transform

## Abstract

**Highlights:**

**What are the main findings?**

- Trend of acoustic emission detection with increasing pipe dent depth analysed in the time–frequency domain.
- Correlation between wavelet coefficient energy and Reynolds number with respect to increasing pipe dent depth.

**What are the implications of the main findings?**

- Utilization of the acoustic emission technique to assess the presence and influence of turbulence within dented pipeline sections.
- The integration of AE signal analysis and CFD modelling offers a promising diagnostic framework for identifying dent mechanisms, assessing damage severity, and enhancing the accuracy of inspection and monitoring strategies in pipeline integrity management.

**Abstract:**

Dents are among the most common deformation defects in buried transmission pipelines, significantly influencing structural integrity and internal flow behaviour. This study examines the occurrence of turbulence in dented pipe sections using time–frequency analysis of acoustic emission (AE) responses. The approach aims to overcome the challenge of obtaining meaningful information from AE signals during conventional dent inspections. By correlating AE spectral characteristics with flow-induced turbulence, the study provides insights into how mechanical deformation influences AE signal behaviour, contributing to an improved assessment of pipeline integrity. In this study, AE signals were captured during flow loop tests on healthy, 5%, 15%, and 30% dented pipe sections to evaluate the influence of dent severity on turbulence behaviour. Time–frequency domain analysis using the Morlet wavelet transform on the starting, middle, and end segments of AE signals revealed a progressive increase in signal energy with increasing dent depth, reaching a maximum of 2.54 × 10^−08^ μE^2^/Hz − 2.54 × 10^−08^ μE^2^/Hz for the end segment of AE signals under the 30% dented pipe condition. Complementary computational fluid dynamics (CFD) simulations were performed to compute velocity streamlines and corresponding Reynolds numbers for validating the turbulence detection results. A strong correlation between the CWT coefficient energy and Reynolds number, with *R*^2^ values of 0.9633, 0.9007, and 0.9052 for the starting, middle, and end signal segments, respectively, was observed. These findings demonstrate that AE time–frequency analysis offers a reliable diagnostic approach for identifying and characterising dent-induced turbulence in pipeline systems.

## 1. Introduction

Thin-walled steel pipelines are extensively deployed in the transportation of crude oil, natural gas, and other petroleum derivatives due to their high efficiency and cost-effectiveness [1]. Nevertheless, their long-term integrity and operational reliability are frequently compromised by aggressive environmental and mechanical conditions. Among the various failure mechanisms, mechanical damage arising from external impact, such as contact with excavation machinery, construction tools, or subsurface rocks, remains one of the most common threats to buried pipeline systems [1,2]. Dents, which represent the most common form of deformation defects, typically result from these impact events, backfill stone indentation, or localised ground settlement [3]. External corrosion, coupled with internal material defects, can further weaken the pipeline structure, potentially leading to catastrophic failure [4].

Moreover, the presence of dents often disrupts protective coating layers, facilitating localised corrosion and accelerating wall thickness reductions. A more critical variant, known as a dent gouge defect, represents a combined form of mechanical indentation and surface gouging that significantly reduces the pressure containment (burst) capacity of the pipeline [4,5]. This type of defect alters both the geometric curvature and local wall thickness, leading to pronounced stress concentration zones and reduced residual strength [5].

Large-diameter gas transmission pipelines, which traverse vast geographical distances to supply consumers, commonly encounter complex geological terrains such as landslide-prone regions, seismic zones, and mined-out areas [6,7,8]. In these environments, soil movement imposes additional bending moments on the pipeline, inducing tensile stress on one side and compressive stress on the other. For pipelines containing pre-existing flaws or dents, these externally induced stresses interact with local geometric irregularities, producing multiaxial stress states that heighten the susceptibility to local buckling and crack propagation [9,10]. Such localised deformation may trigger abrupt instability, reduce structural load capacity, and, in severe cases, lead to leakage or catastrophic rupture. Furthermore, cyclic operational loads, thermal expansion, and pressure fluctuations contribute to progressive degradation, resulting in fatigue-related defects over time [11]. Consequently, an accurate and continuous assessment of pipeline integrity is essential for mitigating risks associated with large-scale pipeline infrastructures.

Although visual inspection remains one of the most widely utilised techniques in structural health monitoring (SHM), it offers limited diagnostic capability. Studies have reported that visual inspection can detect up to 80% of visible surface-level damage, making it a practical and cost-effective method for routine maintenance [12]. It is simple to execute, requires minimal instrumentation, and can be implemented at scheduled intervals. However, its effectiveness is largely restricted to identifying macroscopic surface defects, leaving subsurface or microstructural flaws undetected. Additionally, the technique’s reliability significantly decreases in extensive or inaccessible pipeline networks, such as subsea or buried systems. These limitations render visual inspection unsuitable for continuous or real-time monitoring of structural integrity. In many cases, defects are only detected when they have progressed to a critical stage, undermining the preventive maintenance objectives of integrity management programmes [8].

To overcome these shortcomings, advanced SHM approaches have been explored to enable early detection of structural degradation. Although the conventional practice of the In-Line Inspection (ILI) guidelines from the American Petroleum Institute’s (API) 1163 standard is used to assess dent inspections, limitations still exist in filtering out sensor noise and characterising flaws in dents [13]. Among various non-destructive testing (NDT) techniques, acoustic emission (AE) monitoring has emerged as a promising tool due to its high sensitivity to dynamic damage evolution and capability for real-time assessment. AE-based SHM facilitates the detection of transient elastic waves generated by crack initiation, corrosion activity, or plastic deformation, thereby enabling continuous monitoring of the pipeline’s condition throughout its service life. Guinta et al. conducted a study to monitor pipeline health integrity for a few mechanical damage mechanisms. However, there are still drawbacks in characterising the dent size [14,15,16]. Baensch et al. [17] reported having inspected the bending damage mechanism in the pipeline segment using the AE technique. Meanwhile, Jiao et al. [18] managed to locate leaks on piping using an AE inspection approach. However, these studies only represent the inspection approach for active damage mechanisms instead of existing defect mechanisms, which are more economically crucial, especially in the oil and gas industry. The integration of AE with computational analysis and signal processing methods further enhances its diagnostic potential, offering a reliable pathway toward intelligent, condition-based integrity evaluation of pipeline systems. Therefore, it is necessary to utilise the ability of the AE approach with advanced analysis for identifying existing defects in pipeline applications.

This paper aims to evaluate the AE response toward turbulence flow presence during a flow loop test on pipe sections with various dent sizes and depths. This study utilises a signal processing approach to analyse the AE responses and extract additional information related to the existing damage. To the best of the author’s knowledge, the use of AE time–frequency analysis to detect turbulence effects in dented pipes has not been previously reported. This research is expected to significantly enhance AE-based inspection and monitoring of dent mechanisms in pipeline maintenance.

## 2. Theoretical Background

### 2.1. Acoustic Emission Approach

**Figure 1:**
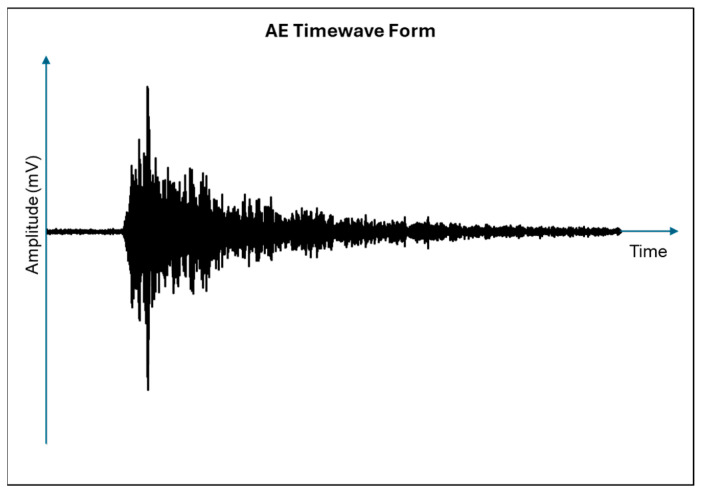
Acoustic emission timewave form signal parameters.

Figure 1 shows the characteristics of an average AE signal. According to Figure 1, amplitude is the highest voltage magnitude that the signal waveform from an emission event can reach during its maximum excursion. It is measured in decibels and represents the highest AE signal excursion during an AE hit, either in a positive or negative value, with a typical AE signal represented as a voltage versus time curve. Voltage is converted to decibels using Equation (1), where *A* is amplitude (dB), *V* is voltage of the peak excursion, and *Vref* is the reference voltage. Commonly, the decibel scale runs from 0 dB (100 µV) to 100 dB (10 mV) [19], with the reference voltage typically being 1 µV.

Figure 1 illustrates the typical characteristics of an average acoustic emission (AE) signal. As shown, the amplitude represents the peak voltage magnitude attained by the AE waveform during its maximum excursion, expressed in decibels (dB). It corresponds to the highest signal excursion of an AE hit, either in the positive or negative direction, with the waveform typically plotted as voltage versus time. The amplitude is calculated using Equation (1), where *A* denotes the amplitude in decibels, *V* is the peak voltage of the signal excursion, and *Vref* represents the reference voltage. In most AE systems, the decibel scale ranges from 0 dB (equivalent to 100 µV) to 100 dB (equivalent to 10 mV), with the reference voltage typically standardised at 1 µV. The amplitude parameter provides a useful measure of the intensity of the emission event and is often used to infer the relative energy released from the source mechanism.

(1) $$A=20\;log\;\frac{V}{V_{ref}}$$

The interval of time between the beginning and end of an AE signal is called the ‘duration’. It is the time interval, measured in microseconds, between the signal’s first and last threshold crossing. The duration of the AE signal has an impact on the threshold level. Signal lengths can also vary throughout AE sources. Electrical pulses and mechanical noise sources are the typical causes of short-duration signals and long-duration signals, respectively. Damage mechanisms can be characterised by duration. It can also serve as a gauge for the severity of the damage [20]. These signals can be captured by strategically placed AE sensors, which convert the mechanical waves into electrical signals for further analysis [21].

### 2.2. Time–Frequency Domain Analysis

The time–frequency domain signal processing method enables the extraction of both temporal and spectral information from a signal, offering advantages over conventional time–domain analysis. In the Continuous Wavelet Transform (CWT), the total signal duration is multiplied by a scale factor that governs the time–frequency resolution. Within this framework, a wavelet function *φ* is defined as a zero-mean function in the function space, expressed as in Equation (2). This condition indicates that the basis function is oscillatory or wave-like in nature [22].

(2) $$\int_{-\infty }^{+\infty }\varphi_{dt=0}$$

A family of wavelets can be generated from the mother wavelet by scaling and shifting, represented as follows:

(3) $$\varphi_{\left(p,q,t\right)}=\frac{1}{\sqrt{p}}\varphi \left(\frac{t-q}{p}\right)$$

where *t* denotes time, *p* represents the scale index, and *q* corresponds to the time-shift parameter. The wavelet coefficient (WC) in the CWT is defined by the following integral:

(4) $${WC}_{\left(p,q\right)}=\frac{1}{\sqrt{p}}\int_{-\infty }^{+\infty }F_{t}\varphi \left(\frac{t-q}{p}\right)dt$$

The analysis begins with a primary function known as the mother wavelet, which serves as the foundation for representing localized signal characteristics in the scale domain. Among various options, the Morlet wavelet was selected because it effectively captures the internal energy distribution patterns that resemble fatigue failure characteristics. The Morlet wavelet, introduced by Morlet et al. [23,24,25], is a complex mother wavelet defined as in Equation (5).

(5) $$\varphi_{\left(p,q,t\right)}=e^{\left[-\frac{-\beta^{2}{\left(t-q\right)}^{2}}{p^{2}}\right]}\cos\frac{\pi \left(t-q\right)}{p}$$

The shape parameter *β* governs the wavelet’s form and maintains a balance between time and frequency resolution. The mother wavelet possesses finite energy, and its internal energy $\bar{e}$ is expressed as follows [26]:

(6) $${\bar{e}}_{\left(p,q\right)}=\int_{-\infty }^{+\infty }{\left|WC_{t}\right|}^{2}dt$$

The wavelet coefficient characterizes the energy distribution of the signal across time and frequency domains, where its squared magnitude represents local signal energy. In acoustic emission (AE) signal analysis, these energy values indicate dynamic amplitude variations. A lower scale corresponds to a higher frequency and smaller amplitude, signifying lower energy cycles with a minimal likelihood of mechanical damage. Conversely, a higher scale represents a lower frequency and larger amplitude, indicating higher energy cycles that are more likely to cause mechanical failure. Thus, low frequencies are typically associated with greater magnitudes, while high frequencies exhibit smaller magnitudes and more frequent occurrences.

### 2.3. Fluid Flow Determination Using Reynolds Number

The Reynolds number is the ratio of inertia forces to viscous forces. It is a dimensionless metric used to group fluid systems where viscosity plays a significant role in regulating fluid velocities or flow patterns. The Reynolds number is expressed as follows:

(7) $$Re=\frac{\rho V_{ch}D_{ch}}{\mu }$$

where *ρ* is density, *V_ch_* is channel velocity, *D_ch_* is the diameter of the inlet channel flow, and *μ* is the viscosity of the fluid. The Reynolds number is used to determine whether a fluid is in laminar or turbulent flow. It is considered that laminar flow is represented by a Reynolds number of less than or equal to 2100, and turbulent flow is represented by a number greater than 2100 [27].

## 3. Results

**Figure 2:**
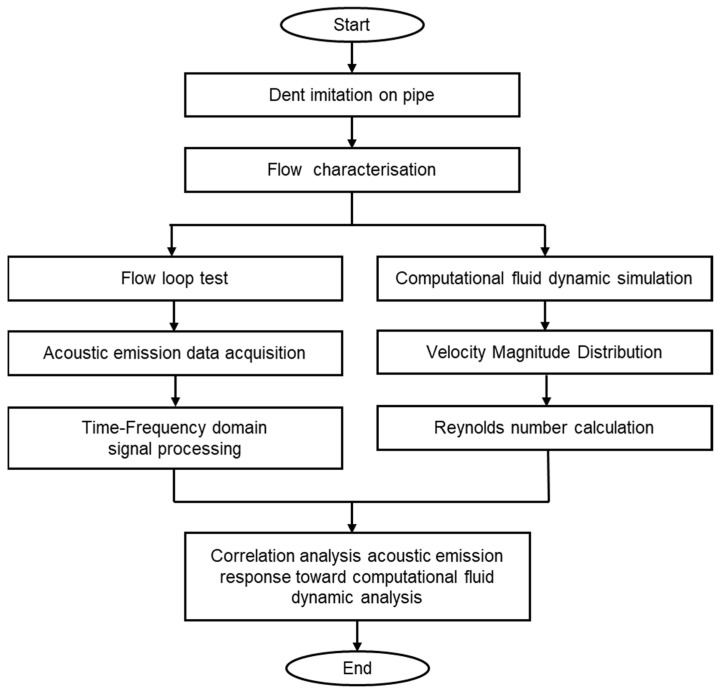
Methodology flow chart for overall work.

This section provides a detailed description of the methodology for this study. The methodology process flow is depicted in Figure 2 to give a clear overview of this work. The methodology included a flow characterisation method through experimental work on the acoustic emission response of dented pipe sections. The post-processing analysis of the acoustic emission response is validated using computational simulations analysis.

### 3.1. Specimen Preparation

For the specimen preparation and testing, the pipe material was carbon steel A105, and the size and length were 4-inch (0.1016 m) schedule 10 and 2000 mm, respectively, as illustrated in Table 1.

**Figure 3:**
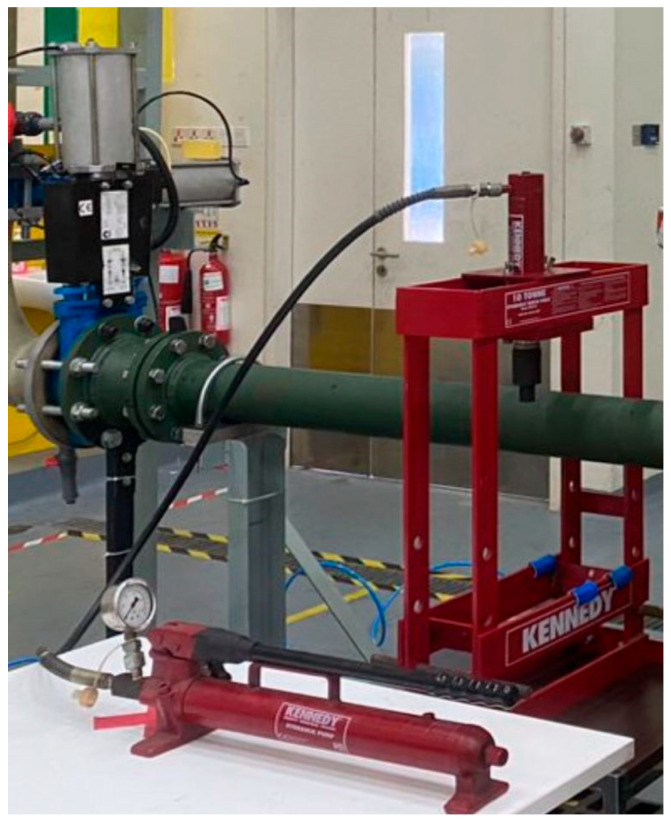
Denting procedure using Kennedy 10 tonne hydraulic bench press machine.

To simulate the exact dent defect in a real situation, the denting process is conducted by manually pressing on the test spool. The pipe spool was dented until it reached a certain depth condition using a 10-tonne capacity hydraulic bench press machine with a flat indenter. To determine the effect of flow hitting at the dented region, the indenter was placed on the middle of the specimen as illustrated in Figure 3. The pipe was subjected to denting to depths of 5%, 15%, and 30% of the outer diameter of the 4-inch (0.1016 m) pipe. The indenter was designed based on the pipe support condition that caused the dent mechanism on the onsite pipeline, with a scaling factor of 6:1 for laboratory test purposes.

Experiments on pipelines, including those that are healthy (undented) and those with dent depths of 5%, 15%, and 30%, are designed to systematically assess how increasing dent severity affects the structural integrity, failure risk, and service life of pipelines. These specific dent depths represent a progression from minor to severe damage, allowing researchers to identify critical thresholds where pipeline performance and safety are significantly compromised. The previous study regarding the designated dent is illustrated in Table 2. The healthy (0%) condition serves as a baseline for comparison, representing undamaged pipe behaviour under load [28]. In contrast, a dent depth of 5% represents minor to moderate damage, which is often considered as being within or near industry-accepted limits for safe operation. Studies show that dents less than 5% of the pipe diameter typically do not pose significant fatigue or burst risks unless subjected to severe cyclic loading [29]. Meanwhile, 15% and 30% dent depths represent increasingly severe damage. These levels are chosen to investigate the transition from moderate to critical damage, where the risk of failure (e.g., burst, buckling, or fatigue) rises sharply. Dents deeper than 10% are often considered critical, and 15% or 30% depths help define the upper bounds of pipeline tolerance and the onset of dangerous failure mechanisms. Testing pipelines at healthy, 5%, 15%, and 30% dent depths enables a comprehensive understanding of how increasing damage affects pipeline safety and performance, helps validate assessment models, and informs industry standards for defect management.

### 3.2. Flow Loop Test Monitoring Using Acoustic Emission Techniques

**Figure 4:**
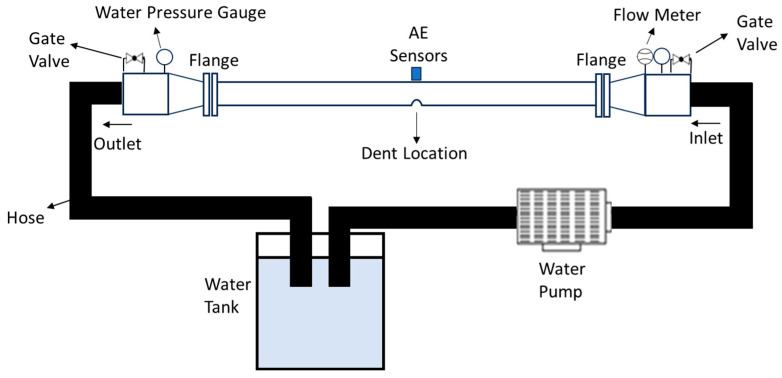
Schematic diagram for flow loop test setup.

The test rig setup was used to verify the aim of this study, as shown in Figure 4. The test setup consisted of 0.1016 m spools filled with water to simulate the flow of the water to hit the dent region of the spools. Water is considered the test fluid because it does not pose any environmental hazards and is safe to use [37]. The piping materials were carbon steel A105, and the size and length were 4-inch (0.1016 m) schedule 10 and 2000 mm, respectively. The head of the centrifugal pump is 15 m, and the volume flow rate is set to 477 L/min.

Furthermore, the data acquisition in this study was performed using AE sensors attached on the top to the spools, as shown in Figure 4. The sampling frequency for the AE data acquisition was set to 1 MHz. Prior to collecting the data, pencil lead break tests were conducted to ensure that the sensors were producing consistent AE signals and responding appropriately. At the beginning of the test, the valve and pump were closed to capture the environment without water flow for AE data acquisition for 2 min. The water flow was then allowed to run under normal conditions, with data capture continuing for 30 min. The test was repeated with a spool at 5%, 15%, and 30% dent depth.

### 3.3. Acoustic Emission Signal Processing in Time–Frequency Domain

Time–frequency domain analysis was conducted using CWT assessment using the Morlet family. This analysis derived based on signal processing as expressed in Equation (5). The selection of the Morlet family of Wavelet Transform is due to its capability in terms of energy representation. It avoids sharp transitions, making it ideal for analysing smooth, continuous, and oscillatory signals such as vibration and fatigue cases, and especially for acoustic emission signals [25]. This approach transforms the signal in the time domain to time–frequency domain, which displays the magnitude distribution at the time and scale axis simultaneously using the AE signal as the data input.

The magnitude distribution level of the wavelet transformation is determined by the colour spectrum contour displayed. The scalogram surface highlights the dominant position and scale in the signal. The scale axis is the inverse of the frequency because at low scales, the compressed wavelets change rapidly, and this usually occurs in high-frequency environments. At high scales, the wavelets are stretched and change slowly for low frequencies. Based on the decomposition of the signal, the energy of the wavelet coefficients can be extracted in each coefficient detail as expressed in Equation (6).

### 3.4. Computational Fluid Dynamics Analysis

**Figure 5:**
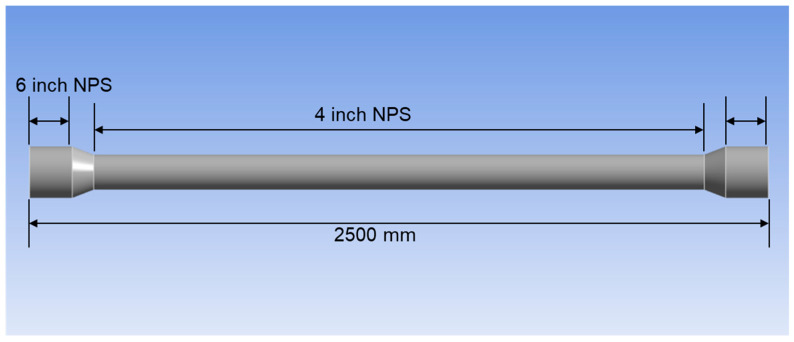
Dimension of modelled pipe.

**Figure 6:**
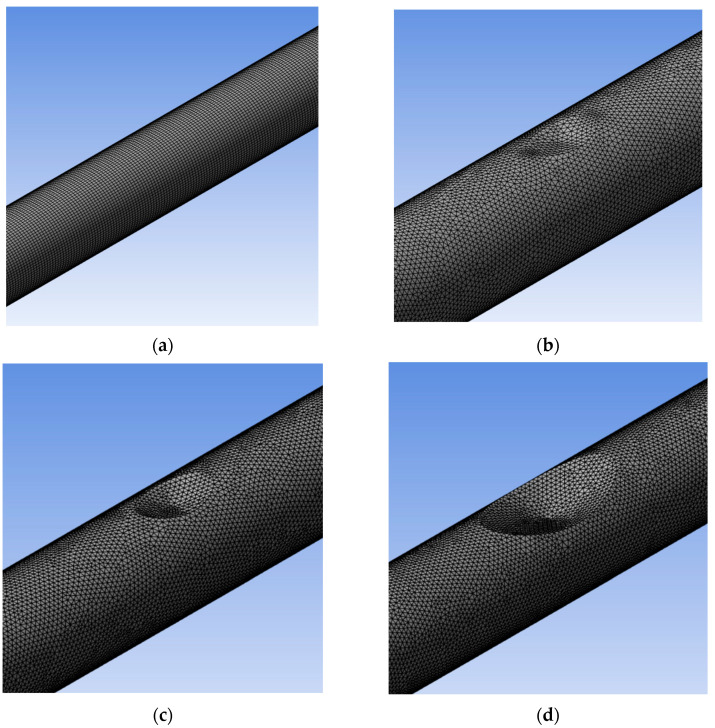
Simulation model with meshing for (**a**) healthy pipe, (**b**) 5% of OD dent pipe, (**c**) 15% of OD dent pipe, and (**d**) 30% of OD dent pipe.

The simulation analysis was done to characterise the flow field distribution within the spool, focusing especially on the dented region. The model geometry was designed with 2.5 m of total length, 2 m for the 0.1016 m spool diameter section, and 0.25 m for the 0.1524 m diameter section on both end sides, as depicted in Figure 5. To the right and left of the model are the inlet and outlet, respectively, and a 0.43 m/s flow velocity was selected based on the flow rate recorded during the flow loop test from the inlet. The models were computed with several dent designs, as illustrated in Figure 6.

## 4. Results and Discussion

This section further explains the acoustic emission signal response from the flow loop test conducted to obtain the induced dented pipe effect. From the data acquisition work, the analysis computed with further signal processing analysis included time–frequency Morlet wavelet and wavelet coefficient energy analysis with validation toward computational fluid simulation analysis.

### 4.1. Acoustic Emission Signal Response on Flow Loop Test

**Figure 7:**
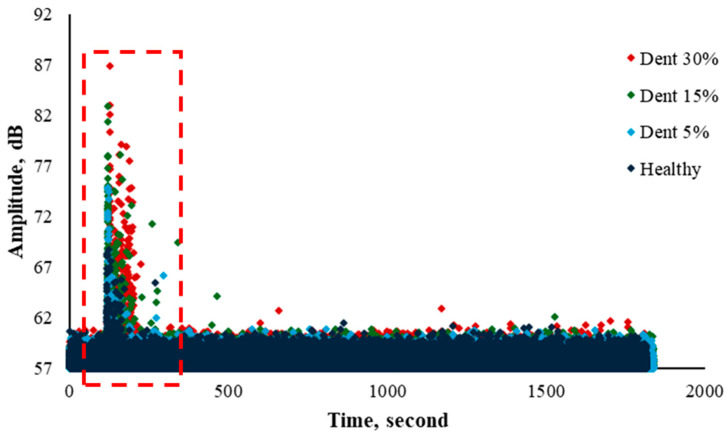
AE signal response toward flow loop test for healthy, 5%, 15%, and 30% OD dent pipe conditions.

The flow loop test was conducted for several pipe conditions: a healthy pipe (with no dent induced) and pipes with dents applied at depths of 5%, 15%, and 30% of the outer diameter (OD) of a 0.1016 m pipe. Figure 7 shows the behaviour characteristics of water that entered the pipe for all these conditions. The threshold was set to 57 dB to eliminate environmental noise. The signal response indicates an abrupt increment at initial data acquisition due to water filling activity between 120 s and 240 s, as highlighted in the red dotted box. In this segment, the AE hits show a linear increment for the maximum hit amplitude recorded from the healthy condition toward 30% of OD dented. When the water-filling activity tends to stabilise within the spool with no bubbles, the AE hit pattern instantly decreases and provides monotonic readings from 240 s until 1800 s for all spool types.

The overall findings show that the responses of AE hits are influenced by the presence of dents. The deeper the dent, the higher the amplitude of AE hits. However, these findings were insignificant due to the slight increment only from a healthy pipe to a 30% OD dented pipe. To enhance this finding, an appropriate advanced signal analysis is required to obtain the hidden information in these AE signals.

Based on the previous studies, the presence and depth of dents can increase AE hit amplitude, but this increase is often modest, especially for shallow or moderate dents, making it difficult to distinguish between healthy and slightly dented pipes using basic AE features alone [38,39].

### 4.2. Time–Frequency Signal Response on Flow Loop of Dented Pipe

**Figure 8:**
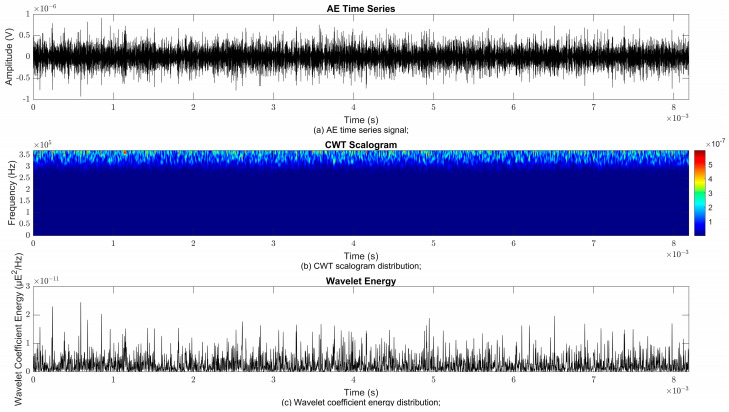
Wavelet coefficient analysis for healthy pipe condition at starting point.

**Figure 9:**
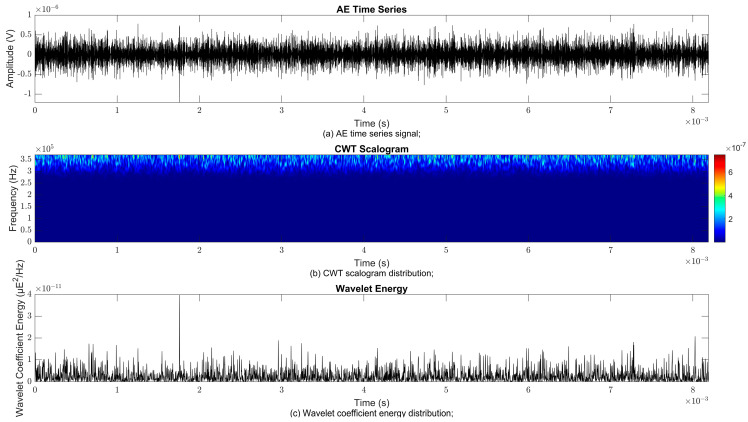
Wavelet coefficient analysis for 5% depth OD at starting point.

**Figure 10:**
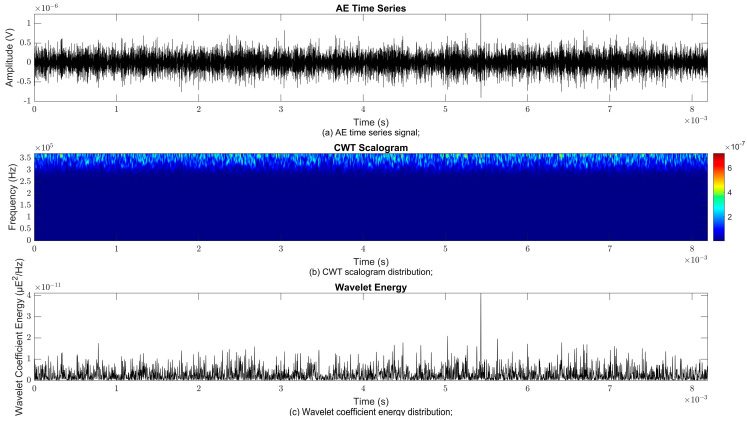
Wavelet coefficient analysis for 15% depth OD at starting point.

**Figure 11:**
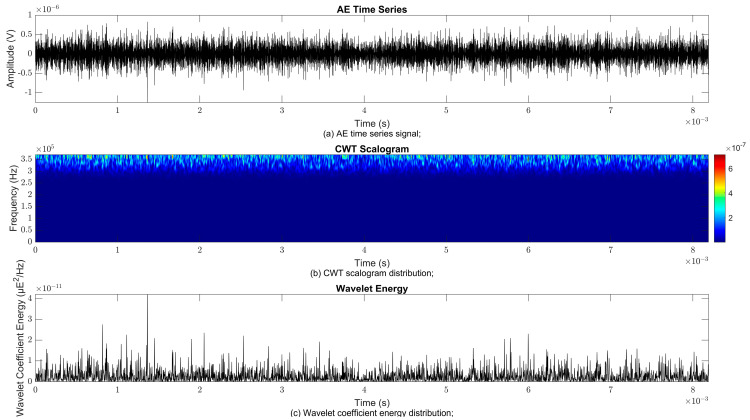
Wavelet coefficient analysis for 30% depth OD at starting point.

**Figure 12:**
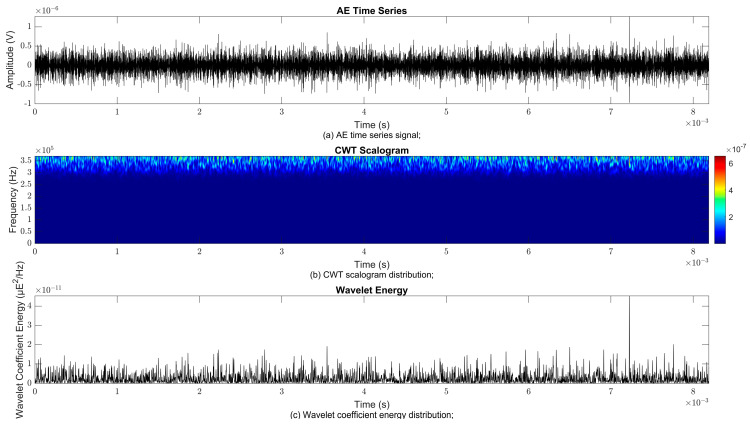
Wavelet coefficient analysis for healthy pipe condition at middle point.

**Figure 13:**
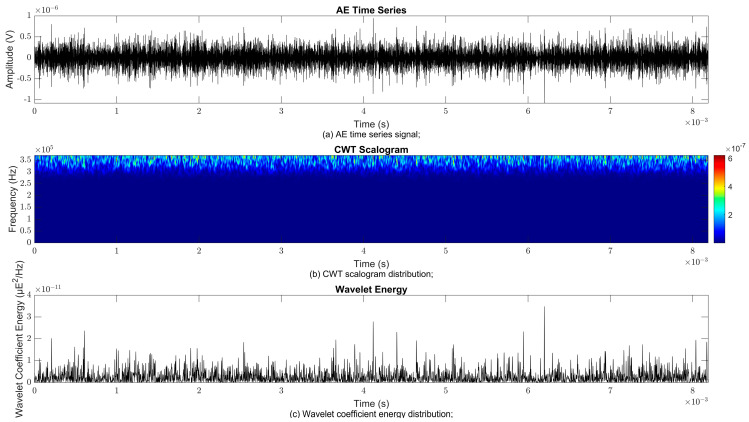
Wavelet coefficient analysis for 5% depth OD at middle point.

**Figure 14:**
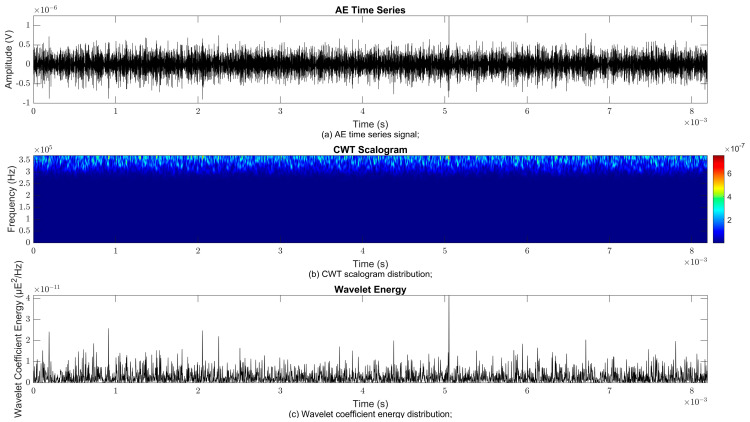
Wavelet coefficient analysis for 15% depth OD middle point.

**Figure 15:**
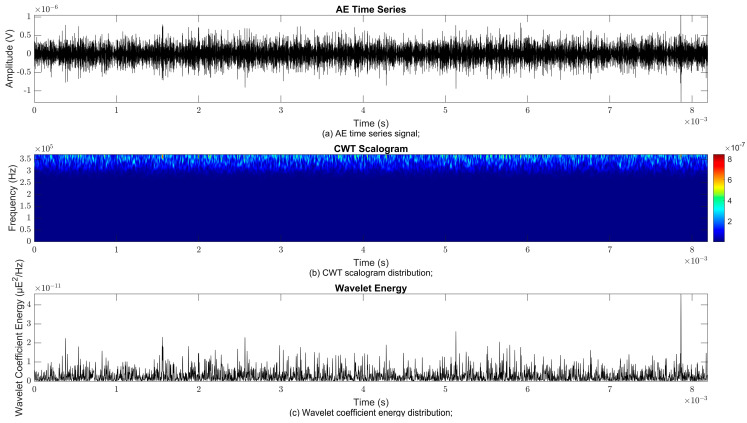
Wavelet coefficient analysis for 30% depth OD middle point.

**Figure 16:**
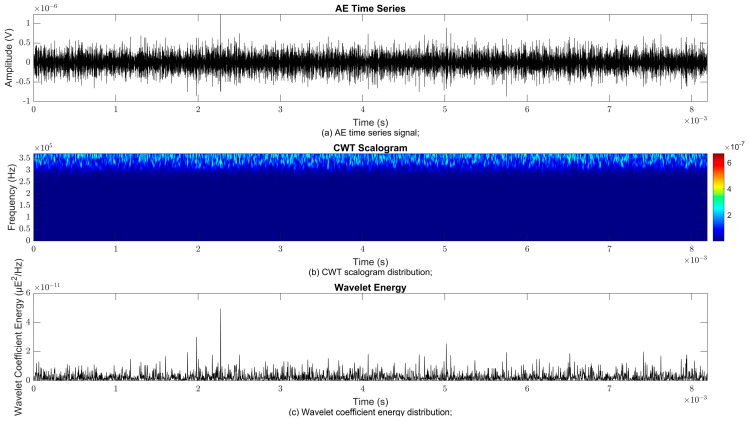
Wavelet coefficient analysis for healthy pipe end point.

**Figure 17:**
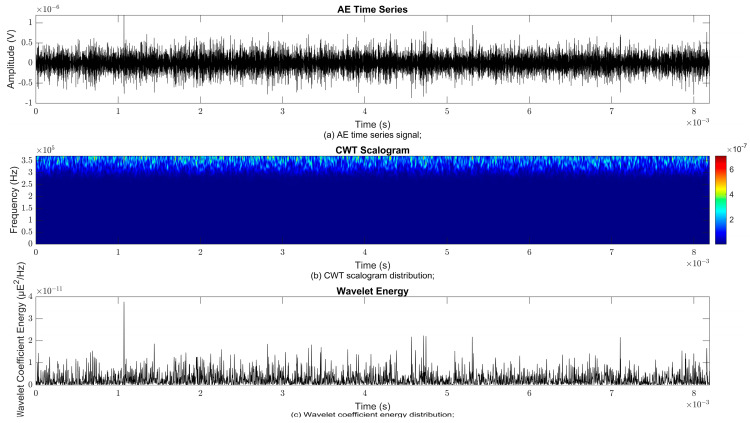
Wavelet coefficient analysis for 5% depth OD end point.

**Figure 18:**
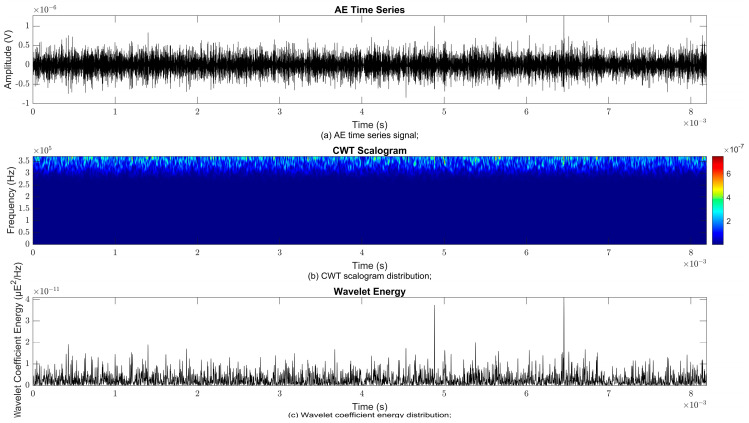
Wavelet coefficient analysis for 15% depth OD end point.

**Figure 19:**
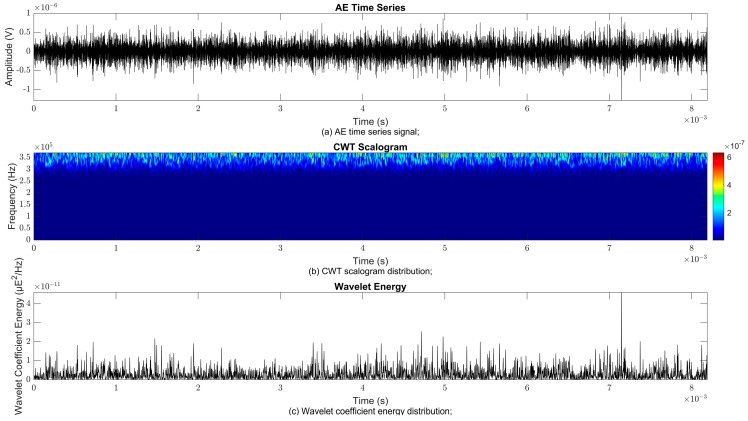
Wavelet coefficient analysis for 30% depth OD end point.

To obtain consistent findings during data acquisition, several points were selected and extracted for further analysis in signal processing; these points corresponded to the starting, middle, and end sections of the acquired AE signal for healthy conditions and depths of 5%, 15%, and 30% of the pipe’s outer diameter. The AE time waveform signal is depicted in part (a), while the processed Morlet wavelet and wavelet coefficient energy are depicted in parts (b) and (c) in Figure 8, Figure 9, Figure 10, Figure 11, Figure 12, Figure 13, Figure 14, Figure 15, Figure 16, Figure 17, Figure 18 and Figure 19. The AE time waveform signal shows similar patterns of continuous form at the starting point for healthy, 5%, 15%, and 30% depths with the signal range of 1.5 × 10^−6^ V to –1.5 × 10^−6^ V. The same goes for all the signal patterns at the middle point and ending point; the trend pattern shows a similar range for all pipe conditions. However, these findings are insufficient to achieve the objective of distinguishing between healthy spool and dented pipe conditions, which is required for enhancement of the time–frequency analysis. The introduction of wavelet coefficient segmentation enhances the accuracy of the segmental wavelet coefficient analysis by limiting every segment to only an overall peak. Therefore, it is more practical to perform wavelet coefficient analysis on segmented data, allowing the coefficients to more accurately represent the localised peakedness of the time series.

The wavelet was applied to change the time–domain signals to time–frequency–domain signals, showing the wavelet coefficient in the time and frequency axes. In the literature, the methodology for choosing the right scale has not been adequately addressed. Hence, a higher scaling factor was applied to acquire better time and frequency resolutions. This is because time resolution is essential for identifying the location of a greater magnitude. Brighter colours imply a high coefficient, while blue colours depict a low coefficient. In Figure 8, Figure 9, Figure 10, Figure 11, Figure 12, Figure 13, Figure 14, Figure 15, Figure 16, Figure 17, Figure 18 and Figure 19, part (b) illustrates the wavelet coefficients, where the matrix rows (*x*-axis) represent the time parameter, while the matrix columns (*y*-axis) denote the scale parameter, which is inversely related to frequency. These coefficients reveal the distribution of AE signal energy within the time–frequency domain [40]. The clustering of the higher wavelet coefficients is within the range of 300 kHz to 350 kHz, as shown in part (b) of Figure 8, Figure 9, Figure 10, Figure 11, Figure 12, Figure 13, Figure 14, Figure 15, Figure 16, Figure 17, Figure 18 and Figure 19. This observation applies for all pipe conditions at the starting, middle, and ending points.

The energy at a given scale and position is expressed through the wavelet energy density function. The corresponding energy spectrum provides essential information regarding the characteristics of the AE signals. As shown in Figure 8b–Figure 19b, the scalogram surface highlights the location of dominant energy concentrations. Energy value in a particular position and scale was represented using a wavelength energy density function. After the energy spectra were developed, it was seen that the load generated vital information related to the pattern of the AE signals. The scalogram area indicated the position of predominant energy, as in part (c) of Figure 8, Figure 9, Figure 10, Figure 11, Figure 12, Figure 13, Figure 14, Figure 15, Figure 16, Figure 17, Figure 18 and Figure 19.

The energy pattern shows an increment in the wavelet coefficient energy trend from the healthy pipe to the 30% depth OD dented pipe. Previous studies in the case of guided wave data show that the reflection coefficient (a proxy for energy in the signal) increases as the depth or size of a defect (such as a dent) grows. The increase in wavelet coefficient energy is due to greater disruption of wave propagation by larger dents, which scatter and reflect more energy back to the sensor. The same situation was expected to occur for the AE data in this case [41,42]. The CWT coefficient energy recorded at the starting point for healthy, 5%, 15%, and 30% OD dented pipe conditions was 2.21 × 10^−8^ μE^2^/Hz, 2.28 × 10^−8^ μE^2^/Hz, 2.36 × 10^−8^ μE^2^/Hz, and 2.42 × 10^−8^ μE^2^/Hz, respectively. For the middle points, the signal also shows a similar increment pattern from healthy, 5%, 15%, and 30% of OD dented pipe with a CWT coefficient energy of 2.23 × 10^−8^ μE^2^/Hz, 2.29 × 10^−8^ μE^2^/Hz, 2.37 × 10^−8^ μE^2^/Hz, and 2.43 × 10^−8^ μE^2^/Hz, respectively.

**Figure 20:**
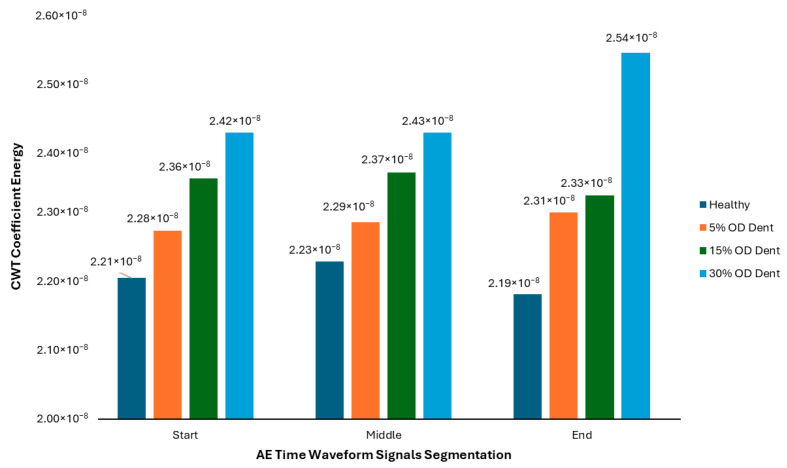
CWT analysis for signals from segmentation.

A similar condition could also be seen in the AE time waveform signal at end points, which were 2.19 × 10^−8^ μE^2^/Hz, 2.31 × 10^−8^ μE^2^/Hz, 2.33 × 10^−8^ μE^2^/Hz, and 2.54 × 10^−8^ μE^2^/Hz for healthy, 5%, 15%, and 30% of OD dented pipe. These findings are further illustrated in Figure 20, where it can be seen that the patterns of the signal show a linear relationship between the response of the AE signal in the time–frequency domain and the pipe dent size. The higher the dent size, the higher the wavelet coefficient of the AE signal response is theoretically. For defects of increasing depth (e.g., from a healthy condition to a 30% OD dent), the energy in the wavelet domain increases, enabling a quantitative assessment of dent severity. The results consistently show that wavelet coefficient energy rises as the dent size or depth increases, reinforcing the potential of wavelet-based analysis for quantitative defect evaluation in pipelines.

### 4.3. Computational Fluid Dynamics Analysis for Flow Loop Simulation

**Figure 21:**
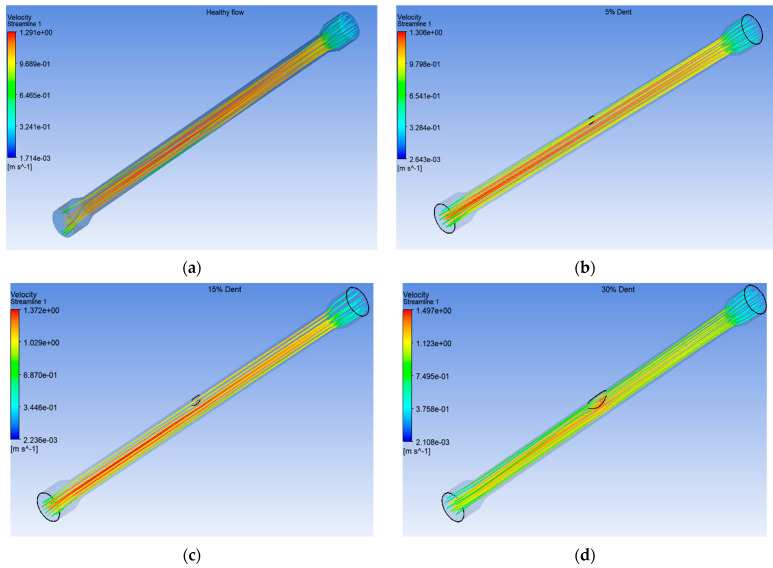
Velocity streamline simulation of (**a**) healthy spool, (**b**) 5% dent, (**c**) 15% dent, and (**d**) 30% dent.

The variation in water flow obtained from the computational fluid dynamics (CFD) simulation illustrates the field distribution, as presented in Figure 21 for healthy, 5%, 15%, and 30% depths of OD dent pipe simulations. The velocity streamline contours indicate that the lowest velocity occurs at the inlet and outlet regions of the 6-inch (0.1524 m) spool, followed by a sharp acceleration as the flow transitions into the 4-inch (0.1016 m) section. Across all the simulated cases, the flow exhibits similar behaviour. However, the healthy pipe condition demonstrates a more uniform and widely distributed velocity field within the flow domain. This observation is consistent with the expected continuity of flow through an undisturbed pipe geometry, where minimal disturbance results in steady-state velocity gradients along the spool length.

**Figure 22:**
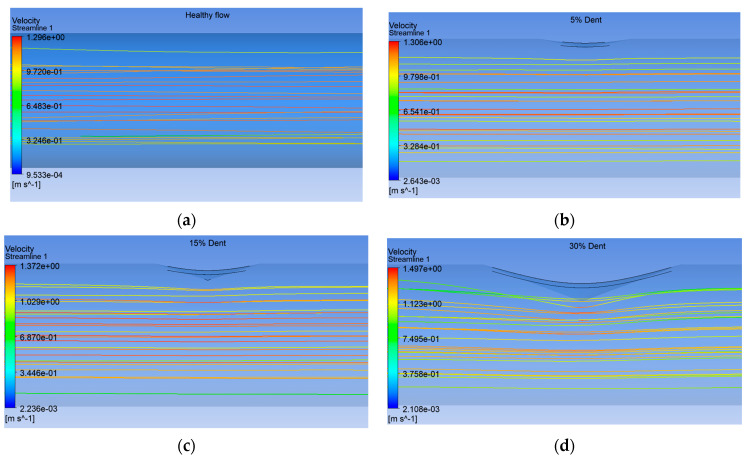
Close view of velocity streamline simulation of (**a**) healthy spool, (**b**) 5% dent, (**c**) 15% dent, and (**d**) 30% dent.

**Figure 23:**
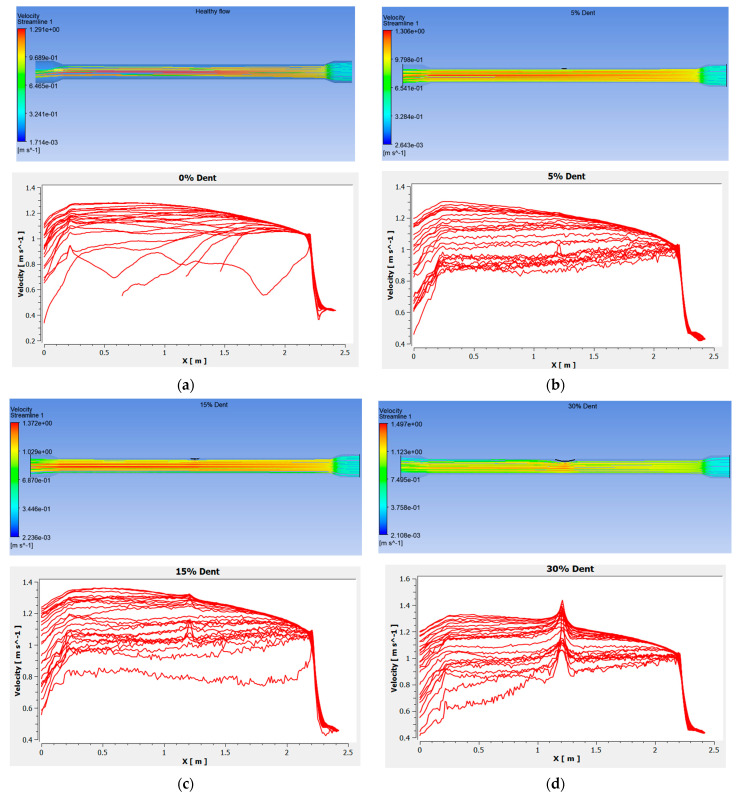
Velocity magnitude distribution distance for the models with the condition of (**a**) healthy spool, (**b**) 5% dent, (**c**) 15% dent, and (**d**) 30% dent.

As shown in Figure 22, the velocity streamline trend exhibits a decreasing pattern with increasing dent depth, from the healthy pipe to the severely dented one at 30% OD in the pipe model. Interestingly, the velocity magnitude exhibits an inverse trend, where the 30% dented configuration records the highest maximum velocity of 1.487 m/s, as depicted in Figure 23. The streamlined profiles further reveal that the dented region induces a localised flow constriction, resulting in an acceleration of the fluid due to the reduction in effective cross-sectional area, in accordance with the Bernoulli and continuity principles. Similar observations have been reported by previous studies on dented or deformed pipe geometries, where local deformation increases the flow velocity and affects the pressure distribution around the defect zone [43,44].

**Figure 24:**
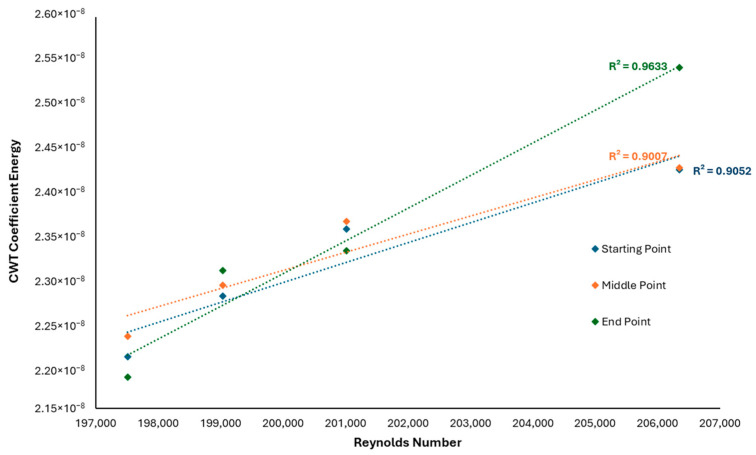
Correlation analysis of CWT coefficient energy toward Reynolds number.

The velocity magnitudes for all configurations were further interpreted as scatter plots, as shown in Figure 24, where the *x*-axis represents the axial length of the spool (with the outlet and inlet positioned on the left and right sides, respectively). The simulated data exhibit a sharp rise in velocity between 2.5 m and 2.3 m for all models, corresponding to the transition from the 0.1524 m to the 0.1016 m section. Within this region, the velocity increases from approximately 0.4 m/s to 1.1 m/s due to fluid regime acceleration through the reducer, which is a characteristic behaviour of converging flow regions in pipeline systems [43].

For the healthy model, the velocity field exhibits more significant fluctuations, indicating natural turbulence associated with the absence of geometrical deformation. In contrast, dented models display smoother velocity transitions, accompanied by a gradual increase in magnitude as the dent depth intensifies. A distinct acceleration zone is observed at the dented region along the spool’s midsection, with the 30% dent case producing the highest velocity spike at 1.487 m/s. Downstream of this region, the velocity rapidly decreases at approximately 0.3 m as the flow expands into the outlet section, transitioning from the 0.1016 m back to the 0.1524 m spool. These findings highlight the strong dependence of local flow behaviour on dent severity and geometry, emphasising its implications on pressure drop, turbulence intensity, and potential fatigue loading within pipeline systems.

### 4.4. Correlation Analysis Between Acoustic Emission Signal Toward Simulation Technique

Table 3 presents the parameters obtained from the analysis to establish correlations among the key findings of this study. The CWT coefficient energy values listed in Table 3 were extracted from the signal acquisition data at three distinct segments, namely the starting, middle, and end of the data acquisition period. In addition, the velocity values were derived from the peak velocity points at the dent regions, as illustrated in Figure 24, for the healthy, 5%, 15%, and 30% dented pipes. These velocity data were subsequently substituted into Equation (3) to compute the Reynolds number, representing the degree of turbulence within the pipe under different dent conditions. The parameters recorded exhibit a generally linear correlation, suggesting consistent trends between flow behaviour and signal response characteristics.

Furthermore, Figure 24 demonstrates the correlation between the CWT coefficient energy of the AE signal responses and the Reynolds number obtained from the computational fluid dynamics simulations of the pipe models. The correlation was quantified using linear regression, yielding coefficients of determination (*R*^2^) of 0.9633, 0.9007, and 0.9052 for the starting, middle, and end segments of the AE signals, respectively. These high *R*^2^ values indicate a strong positive relationship and confirm the suitability of the linear fitting. Although a correlation is typically regarded as excellent when *R*^2^ exceeds 0.9, the present results remain acceptable as they are all above the minimum threshold of 0.8 [45]. The minor deviations observed may be attributed to measurement uncertainties and flow irregularities within the dented regions, which could slightly affect the energy distribution of the AE responses. This is due to the inconsistency of the flow regime pressure generated by the test rig system, but it is still within the range of the required pressure. Nevertheless, the strong correlation supports the hypothesis that turbulence intensity within the fluid flow influences the AE signal characteristics, validating the combined use of CFD and wavelet-based signal analysis for structural integrity assessment.

## 5. Conclusions

In this study, the CWT coefficient energy approach, derived from acoustic emission (AE) signal analysis, was utilised to assess the presence and influence of turbulence within dented pipeline sections under controlled flow loop testing. The experimental programme involved pipe specimens with varying dent severities, which were healthy (no dent), 5%, 15%, and 30% dents based on the outer diameter deformation. The results demonstrated that increasing dent severity led to a proportional rise in AE activity and corresponding CWT coefficient energy, reflecting elevated turbulence intensity and flow perturbation near the dent regions.

Complementary CFD simulations exhibited similar trends, where the local velocity magnitude increased progressively with dent depth, supporting the experimental observations. A linear regression analysis revealed a strong correlation between the CWT coefficient energy of the AE signals and the Reynolds number derived from CFD computations, with coefficients of determination (*R*^2^) of 0.9633, 0.9007, and 0.9052 for the starting, middle, and end signal segments, respectively. These high correlation values indicate a robust linear relationship between AE signal characteristics and flow turbulence parameters.

Overall, the findings substantiate that the AE-based time–frequency analysis using CWT provides a reliable means to quantify turbulence-induced flow behaviour in dented pipelines. For further potential and limitations of this approach, it is recommended to conduct additional testing utilizing cutting-edge technologies to assess the dimensions of current pipeline dents prior to initiating AE data gathering. This information can be cross-referenced and validated to enhance the accuracy of the model and the size of the dent criteria. The integration of AE signal analysis and CFD modelling thus offers a promising diagnostic framework for identifying dent mechanisms, assessing damage severity, and enhancing the accuracy of inspection and monitoring strategies in pipeline integrity management.

## Figures and Tables

**Table 1:** Sample description for the study.

Criteria	Description
Material	Carbon Steel A105
Diameter OD, m	0.1016
Pipe Schedule	10
Length, mm	2000
Nominal Thickness, mm	3.05

**Table 2:** Summary of previous literature on dented pipe cases.

Dent Depth (%OD)	Typical Engineering Significance	Research/Standard Reference	Citation
0 (Healthy)	Baseline, undamaged	Control group	[28,29,30]
5	Minor/moderate, often acceptable	Near industry limits	[29,30,31,32]
15	Severe, approaching critical	Above most standard limits	[29,33]
30	Extreme, well beyond safe limits	Catastrophic failure likely	[34,35,36]

**Table 3:** Correlation parameters based on several dent percentages.

Pipe Condition	CWT Coefficient Energy at AE Signal Segmentation			CFD Simulation Results	
	Starting (μE^2^/Hz)	Middle (μE^2^/Hz)	Starting (μE^2^/Hz)	Velocity (m/s)	Reynolds Number
Healthy	2.21 × 10^−08^	2.23 × 10^−08^	2.19 × 10^−08^	1.296	197,510.4
5% OD dent	2.28 × 10^−08^	2.29 × 10^−08^	2.31 × 10^−08^	1.306	199,034.4
15% OD dent	2.36 × 10^−08^	2.37 × 10^−08^	2.33 × 10^−08^	1.319	201,015.6
30% OD dent	2.42 × 10^−08^	2.43 × 10^−08^	2.54 × 10^−08^	1.354	206,349.6

## Data Availability

The original contributions presented in this study are included in the article. Further inquiries can be directed to the corresponding author.

## Abbreviations

The following abbreviations are used in this manuscript:

AE	Acoustic Emission
CFD	Computational Fluid Dynamic
CWT	Continuous Wavelet Transformation
WC	Wavelet Coefficient
